# Production and Characterization of a Clotrimazole Liposphere Gel for Candidiasis Treatment

**DOI:** 10.3390/polym10020160

**Published:** 2018-02-08

**Authors:** Elisabetta Esposito, Maddalena Sguizzato, Christian Bories, Claudio Nastruzzi, Rita Cortesi

**Affiliations:** 1 Department of Life Sciences and Biotechnology, University of Ferrara, I-44121 Ferrara, Italy; maddalena.sguizzato@student.unife.it (M.S.); nas@unife.it (C.N.); crt@unife.it (R.C.); 2 Antiparasitic Chemotherapy-CNRS 8076, Faculty of Pharmacy, F-92296 Chatenay-Malabry CEDEX, France; christian.bories@orange.fr

**Keywords:** clotrimazole, liposphere, alkyl lactate, xanthan gum, *Candida albicans*, mucoadhesion

## Abstract

This study describes the design and characterization of a liposphere gel containing clotrimazole for the treatment of *Candida albicans*. Lipospheres were produced by the melt-dispersion technique, using a lipid phase constituted of stearic triglyceride in a mixture with caprylic/capric triglyceride or an alkyl lactate derivative. The latter component was added to improve the action of clotrimazole against candida. The liposphere morphology and dimensional distribution were evaluated by scanning electron microscopy. Clotrimazole release kinetics was investigated by an in vitro dialysis method. An anticandidal activity study was conducted on the lipospheres. To obtain formulations with suitable viscosity for vaginal application, the lipospheres were added to a xanthan gum gel. The rheological properties, spreadability, leakage, and adhesion of the liposphere gel were investigated. Clotrimazole encapsulation was always over 85% *w*/*w*. The anticandidal study demonstrated that the encapsulation of clotrimazole in lipospheres increased its activity against *Candida albicans*, especially in the presence of the alkyl lactate derivative in the liposphere matrix. A dialysis method demonstrated that clotrimazole was slowly released from the liposphere gel and that the alkyl lactate derivative further controlled clotrimazole release. Adhesion and leakage tests indicated a prolonged adhesion of the liposphere gel, suggesting its suitability for vaginal application.

## 1. Introduction

*Candida albicans* is a fungus that can locate in different host mucosal surfaces, standing as both a member of the normal microflora (yeast form) and a potential opportunistic pathogen (pseudohyphal form) [1,2]. The potential of *Candida albicans* to colonize various mucosal surfaces highly depends on the presence or absence of members of the normal bacterial microflora. Particularly, the fatty acid environment produced by the host and bacterial microflora can influence and regulate the germination of *Candida albicans* on mucosal surfaces [3,4,5]. The proliferation of *Candida albicans* can generate symptomatic infections, such as vulvovaginal candidiasis, experienced at least once by 75% of women and repeatedly by 6–9% of women [6].

Since a local treatment is the first line of choice in cases of acute vaginal yeast infection, a variety of topical preparations are on the market, mainly containing azole fungistatic agents such as ketoconazole, miconazole, and clotrimazole (CLO) [7]. However, the vaginal administration of these drugs as creams, gels, ovules, and pessaries is often related to some drawbacks, such as leakage of the formulation and low residence time in the vaginal cavity [8]. 

To solve this problem, a bioadhesive formulation should be able to increase the residence time of the dosage form and to enhance its local bioavailability [9,10]. In addition, the inclusion of the antifungal agent into a solid microparticulate system could control its residence time and delivery. 

Among microparticulate systems, lipospheres (LS) represent an interesting choice. LS are microparticles with a solid matrix constituted of lipids, such as triglycerides or fatty acid derivatives, with a mean diameter ranging between 0.2 and 500 μm, where drug molecules can be solubilized or dispersed [11,12,13]. Being constituted of lipids, LS possess attractive properties such as biocompatibility and the capacity to increase the entrapment and bioavailability of poorly water-soluble drug. LS are characterized by good physical stability, low-cost components, ease of preparation and of scaling-up. Because of their properties, LS have been proposed for the delivery of many drugs (e.g.**,** antiinflammatory, antimalarial, antiepilepsy, hypoglycemic, vasodilator, antibiotics, anticancer agents, and vaccines) by oral, cutaneous, subcutaneous, or intramuscular administration [11,12,13,14,15,16,17]. Nonetheless, to our knowledge, LS have never been proposed for vaginal administration. On the basis of this last consideration, in the present investigation, LS were especially designed for CLO delivery on vaginal mucosa to treat *Candida albicans* infection. CLO is widely employed to treat fungal infections topically, indeed oral administration of this active compound is not convenient because of its short half-life and side effects [18]. Since CLO is poorly water-soluble, it requires a proper vehicle to rise the right levels of topical absorption [19]. The lipidic phase of LS was based on stearic triglyceride, a solid lipid commonly employed in foods, and caprylic/capric triglyceride (TRIC) or the lactic acid derivative C_12_-C_13_ alkyl lactate (AL). These liquid auxiliary components have been employed in mixture with stearic triglyceride (TRIST) to possibly modulate and disorganize the solid LS microstructure. Indeed, it has been demonstrated that structured lipid carriers based on mixtures of solid and liquid lipids can encapsulate considerable amounts of drug, controlling its release and expulsion [20,21]. In addition, AL was chosen because of the peculiar antimicrobial activity of lactic acid derivatives, which can exert antifungal effect on the basis of their ability to reduce the pH of the milieu [22]. To obtain adhesive formulations with suitable viscosity for vaginal application, LS were added to a gel constituted of xanthan gum, an anionic polysaccharide [23]. Since polysaccharides are defined as polymeric carbohydrates, xanthan gum can be considered as a natural polymer or a biopolymer [24,25,26]. This polymer is naturally produced by *Xanthomonas campestris* by fermentation to stick the bacteria to the leaves of cabbage-like plants. In chilly water, xanthan gum hydrates rapidly, producing weak gels with shear–thinning properties. Noteworthily, its natural origin assures biocompatibility and biodegradability. Therefore, xanthan gum is widely employed in food products as well as in pharmaceutics as a thickener, stabilizer, and emulsifier. Moreover, it has been recently investigated for the fabrication of matrices with specialized drug release characteristics [25,26]. On the basis of the potential of LS and xanthan gum, in the present study, the association of these components has been proposed to produce a new vaginal delivery system. 

## 2. Materials and Methods 

### 2.1. Materials

The copolymer poly(ethylene oxide) (a)–poly(propylene oxide) (b) (a = 80, b = 27) (poloxamer 188) was a gift of BASF ChemTrade GmbH (Burgbernheim, Germany). Caprylic/capric triglycerides, Miglyol 812 N, (TRIC), was a gift of Cremer Oleo Division (Witten, Germany). C_12_-C_13_ alkyl lactate, Cosmacol ELI (AL), was from Sasol (Milan, Italy). Stearic triglyceride (TRIST), xanthan gum, clotrimazole (CLO), agar, and all other reagents and HPLC solvents were purchased from Sigma-Aldrich, Merck (Darmstadt, Germany).

### 2.2. Methods

#### 2.2.1. Liposphere Production

LS were produced by the melt-dispersion technique [11,12]. Briefly, 1 g of TRIST or a lipidic mixture (reported in Table 1), in the absence or in the presence of 20 mg of CLO, was melted at 75 °C and emulsified with 150 mL of an aqueous phase containing poloxamer 188 (5%, *w*/*w*).

The emulsion was stirred for 1 h at 2000 r.p.m. using a mechanical stirrer Eurostar Digital (IKA Labortechnik, Staufen, Germany) equipped with a three-blade rotor impeller with a diameter of 55 mm. The milky formulation was then rapidly cooled to about 20 °C under stirring in an ice bath, yielding a uniform dispersion of LS. The obtained LS were then washed with water and isolated by filtration through a paper filter. LS were left to dry overnight at 25 °C and weighed.

LS yield was calculated as follows [11]:

% Yield = LS weight × 100/Total weight of lipids employed for LS preparation(1)

#### 2.2.2. LS Morphological and Dimensional Analysis

The morphology of LS was evaluated by variable-pressure scanning electron microscopy (VPSEM) (Zeiss Evo 40XPV, Carl Zeiss AG, Oberkochen, Germany). Briefly, 10 mg of LS were directly put on a stub without any recoat and observed under variable pressure [27]. To analyze the internal morphology, dried LS were sectioned with a long stainless steel blade, under a binocular microscope. LS size distributions were determined measuring at least 300 LS/sample.

#### 2.2.3. CLO Content of LS

The amount of encapsulated CLO per mg of dry LS was determined by disgregating 50 mg of LS in 5 mL of ethanol under stirring (300 r.p.m.) at 60 °C for 2 h. 

The samples were filtered (nylon membrane filters, 0.2 μm pore size, Merck Millipore, Milan, Italy) and analyzed by high-performance liquid chromatography (HPLC) for CLO content, as previously reported [19]. HPLC determinations were performed using a two-plungers alternative pump (Jasco, Tokyo, Japan), an UV-detector operating at 210 nm, and a 7125 Rheodyne injection valve with a 50 μL loop. The samples were loaded on a stainless steel C-18 reverse-phase column (15 × 0.46 cm) packed with 5 μm particles (Hypersil BDS, Alltech, Fresno, CA, USA).

The elution was performed with a mobile phase containing methanol/water 80:20 *v*/*v* at a flow rate of 0.8 mL/min. The retention time of CLO was 6.8 min. 

CLO encapsulation efficiency (EE) was calculated as follows [11]:

EE = amount of CLO detected by HPLC × 100/total amount of CLO employed(2)

All data were the mean of four determinations on different batches of the same type of LS.

#### 2.2.4. Anticandidal Activity Study

The antifungal activity was studied against *Candida albicans* (ATCC 10231). The experiment was performed based on the standardized protocol M27-A2, CLSI. Mother cultures of *C. albicans* strain were set up starting from 1.5 mL aliquots of a liquid nitrogen-stored inoculum put in 250 mL sterile flasks containing 98.5 mL of liquid YEPD medium (yeast extract 0.5%, bactopeptone 1%, glucose 2%; Oxoid, Thermo Fisher Diagnostics, Dardilly Cedex, France), placed at 37 °C on an orbital shaker (110 r.p.m.). The inocula were performed after growth (48 h/35 °C) on Sabouraud dextrose agar. The colonies were suspended in 0.85% sterile saline and this suspension was homogenized in a vortex mixer for 15 s; after that, the cell density was determined in a spectrophotometer, and the transmittance (λ = 530 nm) was adjusted to match the standard 0.5 on the McFarland scale (1 × 10^6^ to 5 × 10^6^ yeast/mL). Subsequently, a 1:50 dilution in RPMI 1640-MOPS-buffered medium was performed, resulting in a final concentration of 1.5 ± 1.0 × 10^3^ yeasts/mL.

The microdilution technique [28,29] was performed in 96-wells polystyrene sterile plates; the culture medium was RPMI1640-MOPS-buffered broth. The tested samples were: LS_TRIST_-CLO, LS_TRIST/AL1_-CLO, LS_TRIST_, LS_TRIST/AL1_, and CLO methanolic solution. Namely, 25 mg of the dry formulation was weighed and suspended in 200 μL of the culture medium in the first well, then two-fold serial dilutions were performed in wells from 1 to 10. For CLO methanolic solution, 100 μL of a CLO solution twice as concentrated as the desired final solution was diluted with 100 μL of culture medium in the first well. 

To each well of the microdilution plate, 100 μL of the standardized inoculum was added. The experiments were run in triplicate. The plates were incubated at 35 °C for 48 h, afterwards 10 μL of 0.5% 2,3,5-triphenyltetrazolium chloride and 10 μL containing menadione 1 mM were added to all wells [21], and the plates were then reincubated at 35 °C for 120 min. After addition of 0.1 mL of acid isopropanol (isopropanol/HCl 1 N, 95:5, *v*/*v*), the plates were placed on a shaker for 5 min to dissolve the formazan crystals. The measurements were performed with a microplate reader at 550 nm, and the minimal inhibitory concentration (MIC) was determined. The statistical analysis was conducted by *t*-Student test.

#### 2.2.5. Gel Production

A weighed amount of xanthan gum (0.5% *w*/*w*) was gradually added to citrate buffer 5 mM, pH 4 (prepared by dissolving citric acid monohydrate and trisodium citrate dihydrate in distilled water) and then mixed for 15 min [9]. The gel was left to stand at 25 °C overnight for complete swelling, afterwards LS (5% *w*/*w*), CLO (0.1% *w*/*w*), or AL (0.05% *w*/*w*) were alternatively added and manually mixed until homogeneous dispersion (gel names and compositions are reported in Table 2). Particularly for the preparation of Gel LS_TRIST_-CLO and Gel LS_TRIST_/_AL1_-CLO, LS_TRIST_-CLO and LS_TRIST_/_AL1_-CLO were respectively added into the xanthan gum gel, while Gel-CLO and Gel_AL1_-CLO, employed as controls, were obtained by directly adding free CLO and AL into the xanthan gum gel.

#### 2.2.6. Viscosity Test

The rheology measurements were performed on Gel_AL1_-CLO and Gel LS_TRIST/AL1_-CLO by a Viscolead ADV, Fungilab viscometer (Fungilab, Barcelona, Spain). The gels were poured into a 250 mL beaker, where they were tested at 25 °C. The spindle was immersed to its immersion mark in the different areas of the beaker, for each trial. The viscosity was measured at different speeds, comprised between 1 and 100 r.p.m.

#### 2.2.7. Spreadability Test

The spreading capacity of Gel_AL1_-CLO and Gel LS_TRIST/AL1_-CLO was evaluated. Namely, after 48 h from preparation, an amount of gel (100 mg) was placed on a Petri dish (3 cm diameter) and pressed by another glass dish with a 500 g mass. The time taken for the gel to fill the entire dish was measured.

The following equation was used for this purpose:

*S* = *m* × *l*/*t* (3)

in which *S* is the spreadability of the gel formulation, *m* is the weight (g) tied on the upper plate, *l* is the diameter (cm) of the glass plates, and *t* is the time (s) taken for the gel to fill the entire diameter [30]. The spreadability test was performed three times, and the mean values ± standard deviations were calculated.

#### 2.2.8. Gel Leakage and Adhesion Test

To test leakage and adhesion of the formulations, citrate buffer pH 4.5 and simulated vaginal fluid (SVF) were prepared [9]. Briefly, to prepare SVF pH 4.5, NaCl, KOH, Ca(OH)_2_, bovine serum albumin, lactic acid, acetic acid, glycerol, urea, and glucose were dissolved in distilled water [9]. Agar (1.5% *w*/*w*) was added to the citrate buffer or SVF and stirred at 95 °C until solubilization. The gels obtained after cooling were then cut to obtain rectangular agar slide. 

The gels LS_TRIST/AL1_-CLO and Gel_AL1_-CLO were colored for the leakage test by dissolving rhodamine (0.05% *w*/*w*) in the gels before adding LS_TRIST/AL1_-CLO or AL and CLO. For the leakage test, 50 mg of colored gels or 2.5 mg of dry LS_TRIST/AL1_-CLO were placed onto one end of a citrate buffer or SVF agar slide. The agar slide was vertically put at an angle of 90° on one of the inner walls of a transparent box, maintained at 37 °C ± 1 °C. The running distance of the gel along the slide was measured 1 and 10 min after the formulation placement. Gel leakage was measured three times, and the mean values ± standard deviations were calculated.

For the adhesion test, 200 mg of Gel_AL1_-CLO and Gel LS_TRIST/__AL__1_-CLO, or 10 mg of dry LS_TRIST/AL1_-CLO were placed at the center of citrate buffer and SVF agar slides. The agar slides were respectively immersed in 10 mL of citrate buffer or SVF at 37 °C ± 1 °C for 2 h. The gel or LS residence times on the slides (adhesion time) were visually compared [31]. The tests were performed three times.

#### 2.2.9. In Vitro CLO Release Studies

The in vitro release studies were performed by dialysis on CLO alternatively included in LS_TRIST/AL1_-CLO, LS_TRIST_-CLO, Gel LS_TRIST/AL1_-CLO, Gel LS_TRIST_-CLO, and Gel_AL1_-CLO [11]. 

Briefly, 200 mg of LS or 4 g of gel were placed into a dialysis tube (molecular weight cut-off 10,000–12,000; Medi Cell International, London, UK), then put into 40 mL of a receiving phase constituted of SVF/ethanol (80:20, *v*/*v*), and shaken in a horizontal shaker (MS1, Minishaker, IKA) at 175 r.p.m. at 37 °C. In addition, the release kinetics of free CLO were investigated, placing in dialysis tubes 2 mg of CLO dispersed in 4 mL of distilled water or in 4 g of xanthan gum gel. Samples of receiving phase were withdrawn at regular time intervals and analyzed by HPLC, as described above. Fresh receiving phase was added to maintain a constant volume. The CLO concentrations were determined six times in independent experiments, and the mean values ± standard deviations were calculated.

The experimental data obtained by the release experiments were fitted to the following semiempirical equations, respectively describing Fickian dissolutive (4) and diffusion (5) release mechanisms [32,33]

*M_t_*/*M*∞ = *K_Diss_ t*^0.5^ + *c* (4)

1 − *M_t_*/*M*∞ = *e^−Kdiff^ t* + *c* (5)

where *M_t_*/*M*∞ is the drug fraction released at the time *t*, (*M*∞ is the total drug content of the analyzed amount of LS), and *K* and *c* are coefficients calculated by plotting the linear forms of the indicated equations. The release data represented by the percentages of released drug (0–8 h) were used to produce theoretical release curves. 

## 3. Results

### 3.1. Liposphere Production and Characterization

A preformulation study was performed to investigate the effect of the lipid composition on LS produced by the melt-dispersion technique [11,12,34]. Particularly, an LS matrix was constituted of sole TRIST or of TRIST in mixture with the liquid auxiliary components TRIC and AL (Table 1). Generally, the yields ranged between 80% and 97% *w*/*w* (Table 3).

**Figure 1:**
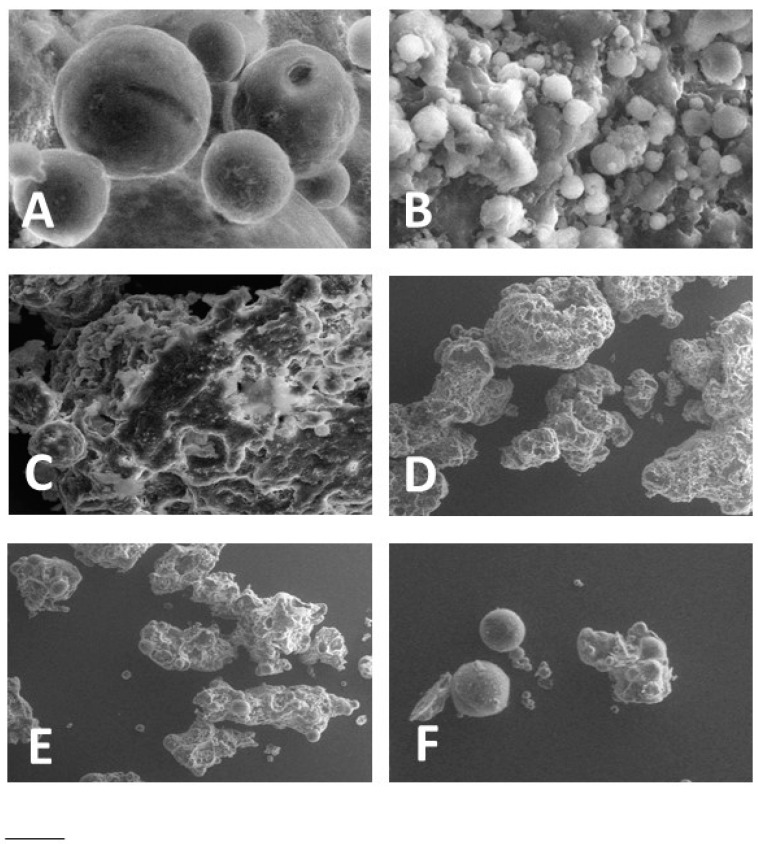
Variable-pressure scanning electron microscopy images (VPSEM) of LS_TRIST_ (**A**), LS_TRIST/TRIC_ (**B**), LS_TRIST/AL30_ (**C**), LS_TRIST/AL15_ (**D**), LS_TRIST/AL10_ (**E**), and LS_TRIST/AL1_ (**F**). Bar represents 20 μm in panels (**A**,**B**), and 50 μm in panels (**C**–**F**). LS acronyms are reported in Table 1.

VPSEM enabled to observe LS characterized by a spheroidal shape, with mean diameters comprised between 6 and 75 μm, and aggregates of LS (Figure 1).

Namely, LS_TRIST_ were spherical, with a 50 μm mean diameter, as shown in Table 3 and Figure 1A. The presence of TRIC (30% *w*/*w*) in mixture with TRIST (LS_TRIST/TRIC_) maintained the LS spherical shape (Figure 1B) and involved a decrease of the LS mean diameter (Table 3). In contrast, the addition of AL (30%, 15%, and 10% *w*/*w*) to TRIST resulted in the formation of collapsed and aggregated LS, with mean diameters difficult to measure (Figure 1C–E, Table 3). A lower amount of AL (1% *w*/*w*) (LS_TRIST/AL1_) resulted, instead, in spherical LS with a 54 μm mean diameter and few aggregates (Figure 1F, Table 3). 

**Figure 2:**
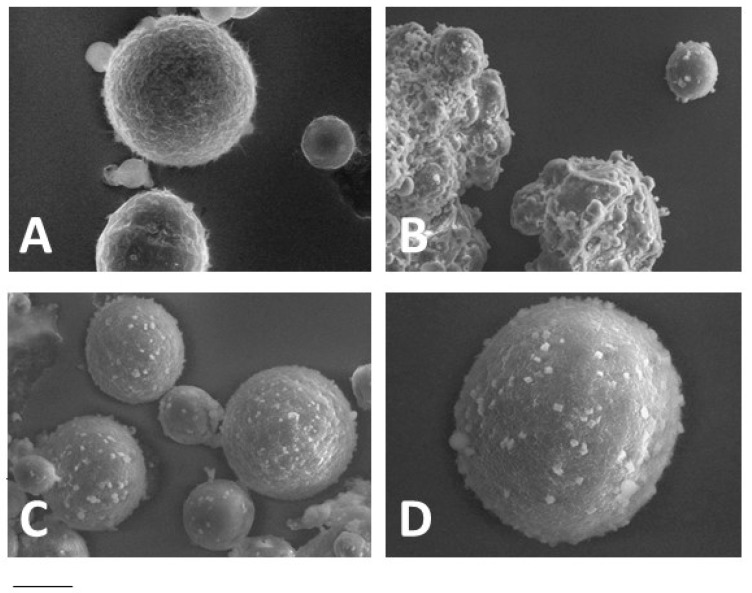
VPSME images of LS_TRIST_-CLO (**A**), LS_TRIST/AL10_-CLO (**B**), and LS_TRIST/AL1_-CLO (**C**,**D**). Bar represents 25, 50, 60, and 15 μm in panels (**A**–**D**) respectively. LS acronyms are reported in Table 1.

LS constituted of TRIST or TRIST/AL mixture (AL 1% and 10% *w*/*w*) were produced in the presence of CLO. The shape and mean diameters of LS_TRIST_-CLO and LS_TRIST/AL1_-CLO were almost unaffected by CLO (Figure 2A,C,D, Table 3), showing a spherical shape and mean diameters around 50 μm, while LS_TRIST/AL10_-CLO were in large part aggregated (Figure 2B). LS_TRIST_-CLO and LS_TRIST/AL1_-CLO were characterized by a matrix-type structure, as it can be observed in Figure S1A,B showing sections of LS obtained by cutting the samples before VPSEM observation. Namely, the inner structure of LS_TRIST_-CLO and LS_TRIST/AL1_-CLO did not show a core–shell organization, suggesting that CLO is probably uniformly dispersed throughout the entire LS.

Regarding CLO EE, the values ranged between 85% and 98%, as reported in Table 1. The highest CLO EE value was achieved in the case of LS_TRIST/AL10_-CLO (98%), nevertheless, this type of LS was discharged because of aggregate formation. 

### 3.2. Anticandidal Activity Study

LS_TRIST_-CLO, LS_TRIST/AL1_-CLO, and the corresponding LS produced in the absence of CLO were assayed against *Candida albicans* (Table 4).

A CLO solution in methanol was employed as a control. CLO-containing LS displayed lower MIC values with respect to the CLO solution (the MIC of LS_TRIST_-CLO and LS_TRIST/AL1_-CLO were, respectively, 1.4 times and 1.9 times lower than the MIC of CLO). In addition, MIC values were lower in the case of LS_TRIST/AL1_-CLO with respect to LS_TRIST_-CLO. The differences between MIC values were statistically significant in the case of LS_TRIST_-CLO versus LS_TRIST/AL1_-CLO (*p* < 0.05) and very significant when both LS were compared with CLO (*p* < 0.01). Empty LS did not display activity, as expected, suggesting that LS did not exert intrinsic antifungal activity [18,35,36].

### 3.3. Production and Characterization of Liposphere Gels

To obtain viscous formulations suitable for vaginal administration, LS, CLO, or AL were included in a xanthan gum gel (Table 2). Since the pH of vaginal hydrogels must be in the range 4–5 [8], we made the choice to use citrate buffer pH 4 instead of distilled water with the aim to prevent pH variation of the formulations, whose pH values were indeed 4.2–4.7. The transparency of the obtained formulations enabled to verify that the LS were homogeneously distributed within the gel.

#### 3.3.1. Gel Viscosity

**Figure 3:**
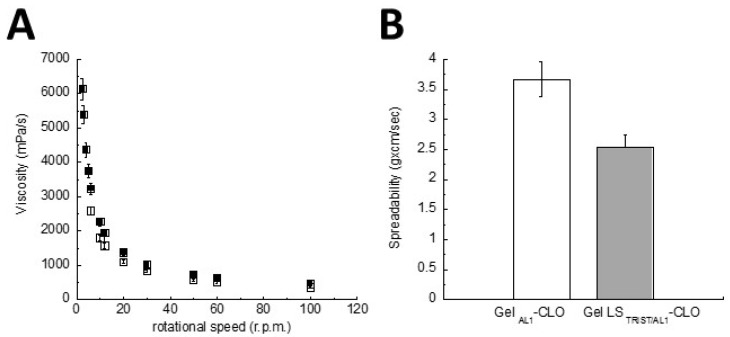
(**A**) Viscosity of Gel LS_TRIST/AL1_-CLO (■) and Gel_AL1_-CLO (□) as a function of the rotational speed; (**B**) Spreadability behavior of the indicated gels. The spreadability was calculated as reported in Section 2.2.7 (Equation (3)). Gel acronyms are reported in Table 2.

A formulation consistency is one of the most important key features for the application on mucosae or skin, thus gel viscosity plays a key role in drug permeation control [37]. The viscosities of Gel LS_TRIST/AL1_-CLO and Gel_AL1_-CLO were, respectively, 661 and 486 mPa/s (25 °C, shear rate 1 s^−1^). The behavior of Gel LS_TRIST/AL1_-CLO and Gel_AL1_-CLO was non-Newtonian shear thinning, indeed their viscosity decreased as the shear rate increased (Figure 3A).

This behavior suggests low flow resistance when applied at high shear conditions [38]. The curves obtained for the plain and LS gels were almost superimposable, indicating that the presence of LS slightly affected the gel viscosity behavior. The symbols on the curves are the means of three experiments, and the error bars represent the standard deviations.

#### 3.3.2. Gel Spreadability

Spreadability is an important parameter for topical forms since it affects patient compliance, extrudability from the package, uniform application on skin or mucosae, dosage transfer, and finally therapeutic efficacy of the active molecule [39]. As expected, LS presence in Gel LS_TRIST/AL1_-CLO slightly decreased the gel spreadability with respect to Gel_AL1_-CLO (Figure 3B). Namely, the spreadability ratio of Gel_AL1_-CLO to Gel LS_TRIST/AL1_-CLO was 1.4:1. 

#### 3.3.3. Gel Leakage

**Figure 4:**
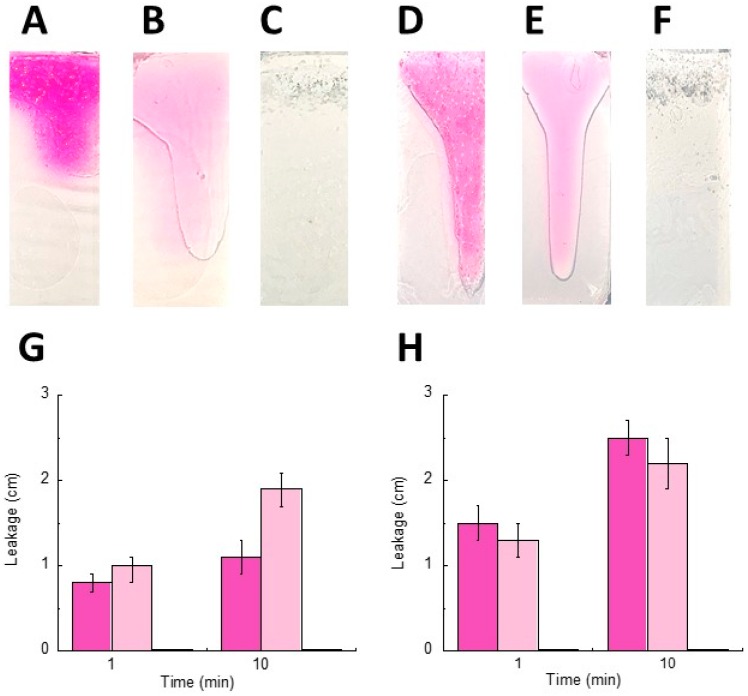
Comparative leakage test performed on formulations colored by rhodamine for imaging. Namely, Gel LS_TRIST/AL1_-CLO (**A**,**D**), Gel_AL1-CLO_ (**B**,**E**), and LS_TRIST/AL1_-CLO (**C**,**F**) were placed on pH 4.5 SVF (**A**–**C**) or citrate buffer (**D**–**F**) agar slides. The leakage distance was measured 1 and 10 min after the application of Gel LS_TRIST/AL1_-CLO (pink), Gel_AL1_-CLO (light pink), and LS_TRIST/AL1_-CLO on pH 4.5 SVF (**G**) or citrate buffer (**H**) agar slides. (**A**–**F**) images were taken 1 h after placing the formulations on the slides. Gel acronyms are reported in Table 2.

The gel leakage potential from the vagina was explored because a vaginal formulation should display a minimal leakage from the vaginal walls and, thus, a short running distance over a vertical plane, to assure a prolonged action [8]. Particularly, the running distances of Gel LS_TRIST/AL1_-CLO, Gel_AL1-CLO_, and LS_TRIST/AL1_-CLO were compared 1 and 10 min after their application on vertical agar slides at pH 4.5, based on SVF or citrate buffer (Figure 4).

The former slides were especially designed to mimic the pH and composition of the vaginal cavity, while the latter ones simulated only the vaginal pH. In the case of the application on SVF slides, the running distance of Gel LS_TRIST/AL1_-CLO was lower than that of Gel_AL1_-CLO, whereas, in the case of the application on citrate buffer slides, the leakage trend was the opposite. Namely, the leakage distance of Gel LS_TRIST/AL1_-CLO applied on cthe itrate buffer slides was almost double with respect to the leakage distance on the SVF slides. This behavior suggests an affinity of Gel LS_TRIST/AL1_-CLO for the SVF slides, and thus for the vaginal fluid composition, rather than for the citrate buffer slides having only the same pH of the vagina. Dry LS remained fixed to the applied site on both type of slides even after 1 hour from placement, suggesting a high affinity for the applied pH and the SVF composition.

#### 3.3.4. Gel Adhesion

**Figure 5:**
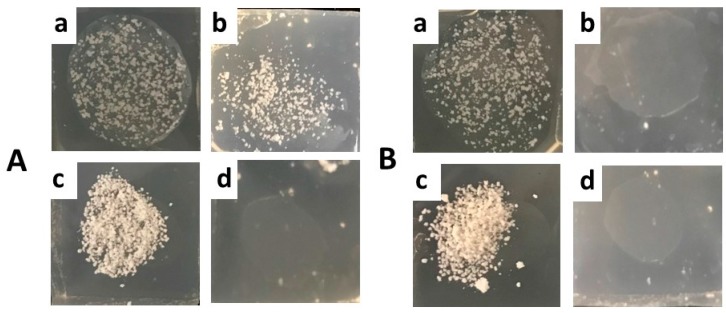
Comparative adhesion test performed on pH 4.5 SVF (**A**) or citrate buffer (**B**) agar plates immersed for 2 h in 10 mL of SVF or citrate buffer, respectively. The images were taken 1 (**a**,**c**) or 120 (**b**,**d**) min after the application of Gel LS_TRIST/AL1_-CLO (**a**,**b**) and LS_TRIST/AL1_-CLO (**c**,**d**). Gel acronyms are reported in Table 2.

Adhesion can be defined as the capability of a material to adhere to a mucosal surface. A high adhesion is required to accomplish the retention of a pharmaceutical dosage form on a mucous membrane [8,9,40]. The adhesion of Gel_AL1_-CLO, Gel LS_TRIST/AL1_-CLO, or dry LS_TRIST/AL1_-CLO was evaluated by comparing their residence times on slides immersed in pH 4.5 SVF or citrate buffer (Figure 5).

After 2 h, the presence of LS was clearly detectable only in the case of Gel LS_TRIST/AL1_-CLO applied on SVF agar slides (Figure 5A, panel b). Indeed, only few LS were detectable in the case of citrate buffer agar slides (Figure 5B, panel b), confirming the suitability of Gel LS_TRIST/AL1_-CLO for vaginal application and contact with vaginal fluids. Two hours after the placement of dry LS on SVF and citrate buffer agar slides, LS_TRIST/AL1_-CLO were almost absent (panels d of Figure 5A,B), suggesting that the inclusion of LS in the gel is required to assure their adhesion. Images of Gel_AL1_-CLO are not reported because the gel instantaneously dissolved after immersion in SVF or citrate buffer, indicating that the LS presence was essential to achieve gel adhesion.

### 3.4. In Vitro CLO Release Kinetics

**Figure 6:**
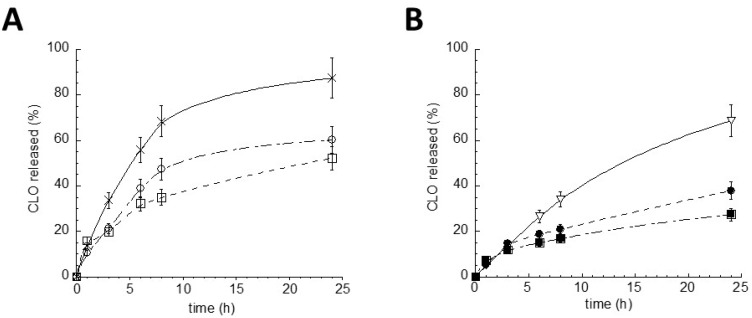
In vitro release kinetics of CLO from LS_TRIST/AL1_-CLO (□), LS_TRIST_-CLO (○) (**A**), Gel LS_TRIST/AL1_-CLO (■), Gel LS_TRIST_-CLO (●) (**B**). The experiments were performed by a dialysis method. For comparison, the profiles obtained using CLO in aqueous (×) (**A**) or xanthan gum (▽) (**B**) dispersions are also reported. The data are the mean of six experiments ± s.d. Gel acronyms are reported in Table 2.

To investigate and compare the performances of Gel LS_TRIST/AL1_-CLO, Gel LS_TRIST_-CLO, LS_TRIST/AL1_-CLO, and LS_TRIST_-CLO as delivery systems for CLO, the release profiles were determined in vitro by a dialysis method [11]. The release kinetics, reported in Figure 6, were in general characterized by a biphasic profile in which CLO was initially released linearly, followed by a slower phase in which the remaining drug was released.

The encapsulation of CLO in LS or in gel enabled to slow down the drug release with respect to the aqueous dispersion (Figure 6A) or the xanthan gum gel (Figure 6B), employed as controls. As expected, the inclusion of LS_TRIST/AL1_-CLO or LS_TRIST_-CLO in gels delayed CLO release. In addition, the presence of AL (1% *w*/*w*) in the LS matrix enabled to better control CLO release, as can be noticed in the LS_TRIST/AL1_-CLO and Gel LS_TRIST/AL1_-CLO kinetics, with respect to their counterparts LS_TRIST_-CLO and Gel LS_TRIST_-CLO (Figure 6A,B). 

To determine the mechanism of CLO release from the studied formulations, a mathematical analysis of the release profile was performed. The theoretical release profiles were calculated according to the linear form of Equations (4) and (5), respectively mimicking dissolutive and diffusive model. Then, a comparison between the theoretical and the experimental release from LS_TRIST_-CLO (Figure S2A), LS_TRIST/AL1_-CLO (Figure S2B), Gel LS_TRIST_-CLO (Figure S2C), and Gel LS_TRIST /AL1_-CLO (Figure S2D) was conducted considering the first 8 h of release. Apart from LS_TRIST_-CLO, for which the experimental curve was almost superimposable to the dissolutive theoretical curve, CLO release from the other formulations was dominated by a mixed release mechanism. Indeed, the experimental curves overlapped partly the theoretical dissolutive curve and partly the theoretical diffusive curve [32,33]. Since LS are characterized by a matrix structure, as demonstrated by VPSEM observation, it can be hypothesized that a slow LS and gel dissolution in contact with vaginal fluids and a dissolution and diffusion of CLO first through LS and then through the gel network take place.

## 4. Discussion

The aim of this study was to verify the anticandidal activity of CLO encapsulated in a LS-based gel and, particularly, in LS constituted of TRIST and AL. This alkyl lactate derivative was added to the LS matrix to possibly improve CLO action. Indeed, it should be considered that CLO, although effective against *Candida albicans* infection, is also destructive to components of the normal vaginal microflora, often leading to an increased risk of infection or disease [41]. Since AL is the ester of lactic acid and a mixture of monobranched C_12_-C_13_ primary alcohols, its hydrolysis produces fatty alcohols and lactic acid [42]. The latter can inhibit bacterial proliferation, as well as the action of lipases that decompose the LS matrix after in vivo administration. Thus, the presence of AL in LS should support CLO activity by controlling bacterial proliferation, re-establishing the pH of the vaginal environment, and modulating LS metabolism. 

The preformulation study enabled to select 1% *w*/*w* as the optimal AL concentration for LS production; indeed higher amounts (10–30% *w*/*w*) hindered LS formation, resulting in collapsed and aggregated LS. The aggregation phenomenon could be attributed to interchain bonds between the AL C_12_-C_13_ alkyl chains present on the surface of this type of LS. Contrarily, TRIC (30% *w*/*w*) led to more regular and structured LS, probably because of the affinity between TRIC and TRIST, both constituted of triglycerides able to give rise to LS by crystallization. As expected, AL presence permitted to increase CLO encapsulation in LS (Table 3). Indeed, it has been demonstrated that a binary mixture of two spatially different lipid matrices, i.e., a solid lipid and a liquid lipid (or oil), results in the formation of structured lipid carriers able to solubilize and encapsulate higher amount of drug with respect to carriers containing a single component [20,21,43].

The anticandidal study indicated, on one hand, that the encapsulation of CLO in LS improved its activity and, on the other, that the combination of CLO and AL in LS further increased the anticandidal activity of CLO, supporting our hypothesis. It can be supposed that the encapsulation of CLO within LS improves its activity because of a close interaction between LS and the fungal cells. It is known that *Candida albicans* adheres to epithelial cells, endothelial cells, soluble factors, extracellular matrix, and inert materials implanted in the body of the host. Indeed, *Candida albicans* adhesion is a prerequisite for colonization and an essential step in the establishment of infection. Namely, the physical interaction between *Candida albicans* cells is mediated by adhesins, the designated cell wall constituents. Thus, it can be reasonably hypothesized that an interaction between the adhesins of the fungal cell wall and the surface of LS occurs, providing a microenvironment able to facilitate CLO release and effect. In the case of the in vitro experiments, in the culture medium, the colonized LS should slowly dissolve and release CLO from their matrix by a dissolution and diffusion mechanism, resulting in a direct close contact of the drug with the fungal cells [44,45]. Conversely, in the case of a CLO methanolic solution, it should be considered that the dilution with the aqueous medium decreases CLO solubility, since CLO is insoluble in an aqueous environment. Thus, the CLO solution would be less efficacious against fungal cells with respect to CLO encapsulated in LS.

Since LS in their dry form cannot be easily applied on mucosae, we included them in a xanthan gum gel and verified the resulting gel suitability for vaginal administration. The obtained results were very encouraging. Particularly, the shear-thinning behaviour of Gel LS_TRIST/AL1_-CLO, whose viscosity decreased when applying a certain force [38], suggests that the gel can be handled and, more importantly, it can easily coat the vaginal cavity, remaining in the application site without draining [8,46], as demonstrated by the leakage experiment.

The leakage and adhesion results agree well, both demonstrating the suitability of the Gel LS_TRIST/AL1_-CLO for vaginal application. In fact, the gel exhibited a minimal leakage and a long adhesion time when applied on SVF agar plates mimicking the vaginal cavity.

Noteworthily, the in vitro dialysis results confirmed that the inclusion of LS in the gel was a successful choice. Indeed, CLO release kinetics were slower in the case of Gel LS_TRIST/AL1_-CLO with respect to the other formulations. The control of CLO release should be attributed to (a) LS matrix, (b) AL presence, and (c) xanthan gum network. Xanthan gum has been described by other authors as a biopolymer able to control drug release. Particularly, it has been employed as a rate-controlling polymer for the development of matrix tablets [25], as a stabilizing agent and delivery vehicle for gold, alginate or iron particles [47,48,49,50], and in combination with other polymers to produce vaginal gels [8,51].

Remarkably, Gel LS_TRIST/AL1_-CLO production can be easily scaled-up for industrial production. Nonetheless, in vivo experiments are required to confirm its suitability to treat vaginal candidiasis.

## Figures and Tables

**Table 1:** Liposphere (LS) composition.

Formulation	Composition (% *w*/*w*)					
	Tristearin (TRIST)	Caprylic/Capric Triglyceride (TRIC)	Alkyl Lactate (AL)	Clotrimazole (CLO)		
LS_TRIST_	100.00		-	-		
LS_TRIST/TRIC_	70.00	30.00	-	-		
LS_TRIST/AL30_	70.00	-	30.00	-		
LS_TRIST/AL15_	85.00	-	15.00	-		
LS_TRIST/AL10_	90.00	-	10.00	-		
LS_TRIST/AL1_	99.00	-	1.00	-		
LS_TRIST_-CLO	98.04		-	1.96		
LS_TRIST/TRIC_-CLO	68.69	29.35	-	1.96		
LS_TRIST/AL30_-CLO	68.69	29.35	-	1.96		
LS_TRIST/AL15_-CLO	83.34	-	14.70	1.96		
LS_TRIST/AL10_-CLO	88.24	-	9.80	1.96		
LS_TRIST/AL1_-CLO	97.06	-	0.98	1.96		

**Table 2:** Gel composition.

Formulation	Gel Components (% *w*/*w*)				
	Tristearin	Alkyl Lactate	Clotrimazole	Xanthan Gum	Water
Gel LS_TRIST_-CLO	4.902	-	0.098	0.500	94.500
Gel LS_TRIST/AL1_-CLO	4.853	0.049	0.098	0.500	94.500
Gel-CLO	-	-	0.098	0.500	99.402
Gel_AL1_-CLO	-	0.049	0.098	0.500	99.353

LS acronyms are reported in Table 1.

**Table 3:** Liposphere mean diameter, yield, and clotrimazole encapsulation efficiency.

Formulation	Mean Diameter (μm)	Yield (%) ^a^	CLO EE (%) ^b^
LS_TRIST_	50 ± 28	92.0 ± 1	-
LS_TRIST/TRIC_	6.3 ± 8	88.3 ± 2	-
LS_TRIST/AL30_	n.d. *	80.0 ± 1	-
LS_TRIST/AL15_	n.d. *	86.0 ± 2	-
LS_TRIST/AL10_	n.d. *	89.7 ± 3	-
LS_TRIST/AL1_	54.2 ± 30	93.3 ± 2	-
LS_TRIST_-CLO	55.2 ± 10	87.0 ± 8	85 ± 7
LS_TRIST/AL10_-CLO	48.2 ± 7	88.2 ± 5	98 ± 2
LS_TRIST/AL1_-CLO	63.4 ± 9	97.8 ± 2	90 ± 8

^a^ LS weight × 100/total weight of lipids employed for LS preparation; ^b^ amount of CLO detected by HPLC × 100/total amount of CLO employed; * not determined; LS acronyms are reported in Table 1.

**Table 4:** Minimum inhibitory concentration values (MIC, ng/mL) of clotrimazole-loaded lipospheres against *Candida albicans* ATCC 10231.

Formulation	MIC (ng/mL) ± s.d. ^a^
LS_TRIST_-CLO	23 ± 1.6
LS_TRIST/AL1_-CLO	17 ± 1.4
LS_TRIST_	no activity
LS_TRIST/AL1_	no activity
CLO	32 ± 2.3

^a^ Standard deviation; LS acronyms are reported in Table 1.

## Supplementary Materials

The following are available online at http://www.mdpi.com/2073-4360/10/2/160/s1, Figure S1: Variable pressure scanning electron microscopy images of LS_TRIST_-CLO (A) and LS_TRIST/AL1_-CLO (B), Figure S2: Comparison of the theoretical (x) and experimental CLO profiles from LS_TRIST_-CLO (A), LS_TRIST/AL1_-CLO, (B), Gel LS_TRIST_-CLO (C) and Gel LS_TRIST /AL1_-CLO (D), The theoretical curves were obtained using the coefficient calculated by linear regression of the linearized form of Equation (4) (□) and Equation (5) (○).

## Abbreviations

LS	Liposphere
AL	C_12_-C_13_ alkyl lactate
TRIST	stearic triglyceride
TRIC	caprylic/capric trigliceride
CLO	clotrimazole
VPSEM	variable-pressure scanning electron microscopy
MIC	minimal inhibitory concentration
