# Supplementary material for: Production and Characterization of a Clotrimazole Liposphere Gel for Candidiasis Treatment
Source: Polymers (Basel). 2018 Feb 8;10(2):160. doi: 10.3390/polym10020160 (PMC6414984; doi:10.3390/polym10020160)
Supplement: Supplementary file 1 [file polymers-10-00160-s001.pdf]

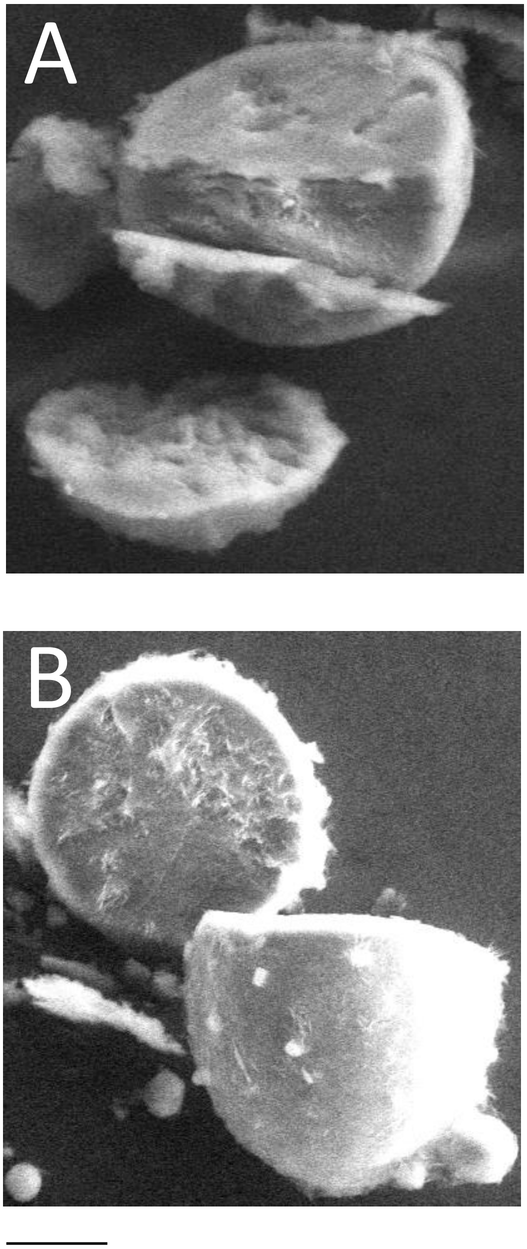

Figure S1: Variable pressure scanning electron microscopy images of LS_{TRIST}-CLO (A) and $LS_{TRIST/AL1}$-CLO (B). Bar represents 20 μm.

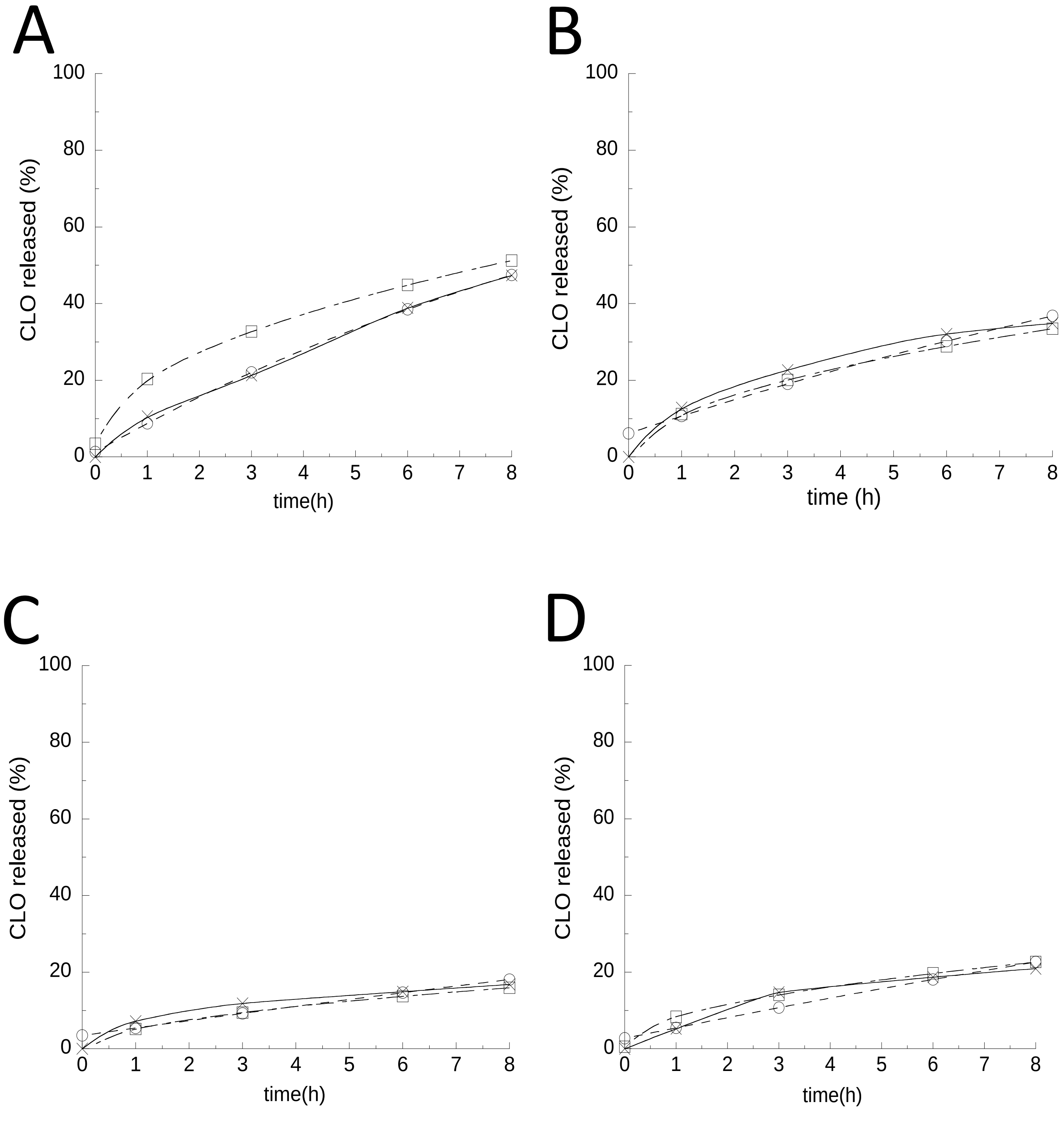

Figure S2: Comparison of the theoretical (x) and experimental CLO profiles from LS_{TRIST}-CLO (A), $LS_{TRIST/AL1}$-CLO, (B), Gel LS_{TRIST}-CLO (C) and Gel $LS_{TRIST/AL1}$-CLO (D), The theoretical curves were obtained using the coefficient calculated by linear regression of the linearized form of equation (4) (□) and equation (5) (○).
